# Effects of Creatine Supplementation and Resistance Training on Muscle Strength Gains in Adults <50 Years of Age: A Systematic Review and Meta-Analysis

**DOI:** 10.3390/nu16213665

**Published:** 2024-10-28

**Authors:** Ziyu Wang, Bopeng Qiu, Ruoling Li, Yunzhi Han, Carl Petersen, Shuting Liu, Yinkai Zhang, Chang Liu, Darren G. Candow, Juan Del Coso

**Affiliations:** 1 China Swimming College, Beijing Sport University, Beijing 100084, China; 2021210263@bsu.edu.cn (Z.W.);; 2 School of Strength and Conditioning Training, Beijing Sport University, Beijing 100084, China; qiubopeng@bsu.edu.cn (B.Q.);; 3 School of International Chinese Language Education, Beijing Normal University, Beijing 100875, China; 4 Faculty of Health, University of Canterbury, Christchurch 8041, New Zealand; carl.petersen@canterbury.ac.nz; 5 China Wushu School, Beijing Sport University, Beijing 100084, China; 6 School of Sport Science, Beijing Sport University, Beijing 100084, China; 7 Faculty of Kinesiology and Health Studies, University of Regina, Regina, SK S4S 0A2, Canada; darren.candow@uregina.ca; 8 Sport Sciences Research Centre, Rey Juan Carlos University, 28943 Fuenlabrada, Spain

**Keywords:** ergogenic aids, performance enhancement, sports nutrition, muscle performance, 1 RM

## Abstract

Background: Numerous meta-analyses have assessed the efficacy of creatine supplementation in increasing muscle strength. However, most have not considered the effect of the participants’ age, training duration, or other confounding variables on strength outcomes. Therefore, the purpose of this study was to consider the effect of these variables on the potential efficacy of creatine supplementation and resistance training for improving measures of muscle strength. Methods: Four databases were searched (MEDLINE, Scopus, Embase, and SPORTDiscus) with a search end date of 22 May 2024. Twenty-three studies were included, with 20 studies involving males (447 male participants), 2 studies involving females (40 female participants), and 1 study involving both males and females (13 male participants and 9 female participants). Results: In comparison with a placebo, creatine supplementation combined with resistance training significantly increased upper-body (WMD = 4.43 kg, *p* < 0.001) and lower-body strength (WMD = 11.35 kg, *p* < 0.001). Subgroup analyses showed a trend for greater upper-body strength improvements for males on creatine compared with females on creatine (*p* = 0.067, Q = 3.366). Additionally, males who consumed creatine combined with resistance training significantly increased both upper- and lower-body strength, whereas females showed no significant gains. There was a trend indicating greater lower-body strength gains from high-dose creatine compared with lower doses (*p* = 0.068, Q = 3.341). No other variables influenced the effect of creatine supplementation. In conclusions, creatine supplementation with resistance training enhances upper- and lower-body muscle strength in adults aged < 50, with greater benefits likely to be seen in males than females.

## 1. Introduction

Creatine supplementation is one of the most commonly used ergogenic aids by individuals involved in high-intensity and/or strength-based sports and activities (e.g., resistance training) [1,2]. It is well established that creatine supplementation, primarily when combined with resistance training, increases measures of muscle performance, specifically muscle strength, across a variety of populations [3]. In physiological and functional terms, creatine supplementation may augment muscle strength by increasing intramuscular creatine stores (phosphocreatine (PCr) and free creatine), which may help resynthesize ATP during and following intense muscle contractions [4]. Creatine has also been shown to increase GLUT-4’s transport kinetics, which could increase blood glucose disposal within the muscle and increase glycogen resynthesis [4]. Finally, creatine reduces blood acidosis and suppresses H⁺ formation during exercise [5], which may allow an individual to perform more repetitions during each set, leading to greater muscle strength over time. These adaptations not only enhance muscle performance during acute high-intensity exercise but also result in greater strength gains when creatine is consumed over a prolonged period in conjunction with resistance training, compared with resistance training alone [3].

In recent years, several reviews and meta-analyses have summarized the wide number of randomized controlled trials (RCTs) involving creatine supplementation on muscle performance variables [6,7,8,9,10]. Collectively, the results have shown that creatine supplementation leads to greater improvements in muscle strength compared with a placebo. However, the generalizability and/or conclusions of these publications are limited in that they included various forms of exercise training (i.e., resistance training, aerobic, weight-bearing) involving participants across a wide age range with varying fitness levels. In this context, several reviews and meta-analyses have investigated the combined effects of creatine supplementation and resistance training—an effective intervention for increasing or maintaining muscle strength—on strength in the older population [11,12]. These studies have focused on the benefits of creatine for older adults, who are more susceptible to muscle mass and strength loss as a result of aging. Collectively, these studies have suggested that a combination of creatine supplementation and resistance training results in greater gains in muscle strength compared with resistance training and a placebo among older individuals. Moreover, it has been shown that creatine supplementation may be more effective in studies including participants > 50 years of age, as creatine supplementation may counteract the age-related reduction in muscle strength [3,11,12]. Interestingly, only one recent meta-analysis focused on the effect of creatine supplementation on fat mass in younger adults (under 50 years of age) has been published [13]. Consequently, the effect of creatine supplementation on strength gains from resistance training in adults under 50 years of age remains unknown to date.

Another limitation of the existing literature on this topic is the method of data pooling, which partially restricts the applicability and clarity of the results. The abovementioned meta-analyses have predominantly examined the pooled effect of creatine supplementation on muscle strength using standardized mean differences (SMD). However, interpreting the overall effect of creatine supplementation on muscle strength can be challenging, since SMD presents its effect size hierarchy in units of standard deviation rather than in more applicable units such as kilograms. Therefore, a more specific systematic review and meta-analysis is needed to update the outcomes of studies on the effect of creatine supplementation and resistance training on muscle strength performance, by using more severe filters to assess the magnitude of the effect on participants < 50 years of age. In line with this objective, a systematic review and meta-analysis were conducted of all RCTs that compared a group of healthy adults < 50 years of age receiving creatine supplementation during a controlled resistance training protocol vs. a comparable group of adults receiving placebo supplementation during the same resistance training protocol. Additionally, the presentation of the studies’ pooled data focused on calculated improvements in muscle strength with creatine with respect to the placebo, measured in kilograms of gain. It was hypothesized that creatine supplementation combined with resistance training would enlarge muscle strength gains in comparison with the combination of a placebo and resistance training in adults < 50 years of age.

## 2. Methods

### 2.1. Search Strategy

This systematic review was conducted in accordance with the PRISMA guidelines for Exercise, Rehabilitation, Sports Medicine, and Sports Science (PERSiST) 2020 [14] and registered on the International Prospective Register of Systematic Reviews (PROSPERO) (ID: CRD42024464243). A comprehensive search was performed using relevant keywords related to creatine supplementation and resistance training, using both Medical Subject Headings (MeSH) and free text words. The databases used for the search included MEDLINE (via PubMed), Scopus, Embase, and SPORTDiscus (via EBSCO). The search was conducted from the inception of the database until 22 May 2024. The search strategy utilized key concepts: Concept 1 (strength training OR resistance training) AND Concept 2 (creatine monohydrate supplementation OR creatine supplementation). All titles and abstracts reported in these searchers were cross-referenced manually using Endnote 20 (Clarivate Analytics, London, UK). Subsequently, the titles and abstracts were carefully examined to detect duplicates and to narrow down the relevant studies for the full-text review. Two independent reviewers (Z.W. and B.Q.) conducted the searches, resolving any discrepancies through discussion and consensus. An a priori analysis of the inter-rater reliability showed that these reviewers had a kappa score of 0.83 regarding the number of studies found in each database.

### 2.2. Eligibility Criteria

The inclusion criteria for the reviewed studies were established on the basis of the PICOS principle (Population, Intervention, Comparison, Outcome, and Study design). The review was tailored to include only RCTs that compared the combination of creatine supplementation and resistance training with placebo supplementation and resistance training. Additionally, only RCTs focusing on outcomes related to muscle strength measured before and after resistance training were included. Only RCTs with healthy individuals and resistance-trained individuals < 50 years of age were included to help decrease the influence of biological aging on muscle strength (i.e., sarcopenia/dynapenia). Last, systematic reviews and meta-analyses were excluded, as well as studies that were not available in full text, acute interventions, non-peer-reviewed articles, opinion pieces, reviews, case reports, and editorials. Table 1 presents a detailed overview of the inclusion criteria.

### 2.3. Assessment of the Methodological Quality of the Included Studies

Two authors (Z.W. and B.Q.) independently assessed the methodological quality (i.e., Items 2–9) of the included studies using the Physiotherapy Evidence Database (PEDro) scale [15]. Any disagreement between researchers was resolved by consensus. Studies were scored as excellent (score: 9–10), good (score: 6–8), fair (score: 4–5), or poor (score: < 4) [16]. The score across all studies was 7.00 ± 0.95, indicating that the overall quality of the articles was categorized as “good”. An a priori analysis of inter-rater reliability indicated that the reviewers achieved a kappa score of 0.91 for the scores of the included studies using the PEDro Scale. A more detailed explanation of the score obtained by each study is presented in the results section.

### 2.4. Data Extraction

The following characteristics of the included studies were extracted: (1) research characteristics (authors, year of publication, and country), (2) participants’ characteristics (sample size; age, sex, and training status), (3) creatine supplementation characteristics (dose, duration of creatine loading (if any), and duration of creatine maintenance), and (4) resistance training characteristics (frequency and duration of the intervention). The primary outcome of this review was the effect of creatine supplementation on maximal muscle strength, and the data were extracted from tests that measured this variable before and after the resistance training program, mainly with one-repetition maximum tests (1 RM). For each of the groups included in each study, the sample size was included in the analysis, and the means and standard deviations were extracted for both the pre- and post-intervention measurements for the creatine and placebo groups. For studies that did not provide detailed information on the pre- and post-intervention measurements for the creatine and placebo groups but met the inclusion criteria for this systematic review, the authors were contacted by email to obtain such data.

### 2.5. Data Analysis

Comprehensive Meta-Analysis software (version 4; Biostat, Englewood, NJ, USA) was used as the data analysis processing software for this review. For the meta-analyses, weighted mean differences (WMD) were calculated, and 95% confidence interval (CI) estimates were obtained from studies by comparing pre–post-training changes in muscle strength in the creatine and placebo groups. WMD was used to provide the effect of creatine supplementation over the placebo in kilograms, as mentioned above. The *I*^2^ statistic was used to measure the degree of heterogeneity, with values less than 50% indicating low heterogeneity, 50–75% indicating moderate heterogeneity, and values greater than 75% indicating high heterogeneity [17,18].

In the subgroup analyses, the effects of creatine supplementation on muscle strength were examined, depending on (1) the characteristics of creatine supplementation (low-dose: ≤5 g/day vs. high-dose: >5 g/day [19]; creatine loading phase vs. no creatine loading loading), (2) the characteristics of resistance training (training ≤3 times per week vs. >3 times per week; intervention period <8 weeks vs. ≥8 weeks), and (3) population characteristics (resistance-trained vs. untrained; males vs. females). To assess potential publication bias in the combined data from each study, funnel plots were visually examined, and Egger’s linear regression tests were used. [20]. Moreover, Duval and Tweedie’s trim and fill method was used to identify missing studies if there was a potential publication bias [21]. Statistical significance was considered at *p* < 0.050.

## 3. Results

### 3.1. Study Selection

**Figure 1:**
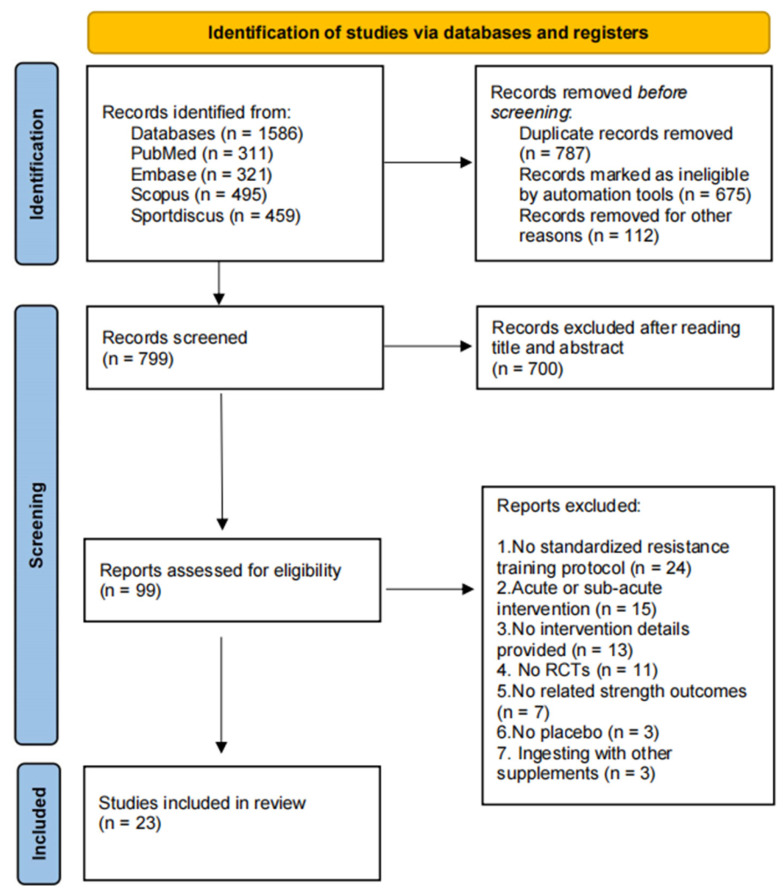
Literature search flowchart, following the PRISMA 2020 guidelines.

After removing duplicates, 799 records remained from the initial 1586 found during the original search. Following title and abstract screening, 700 records were excluded, leaving 99 articles for full-text examination. Ultimately, according to the studies’ characteristics, 23 papers [22,23,24,25,26,27,28,29,30,31,32,33,34,35,36,37,38,39,40,41,42,43,44] were selected for the current systematic review and meta-analysis, while the remaining 76 were discarded (Figure 1).

### 3.2. Methodological Quality of the Studies

Using the PEDro scale, it was determined that of the 23 studies [22,23,24,25,26,27,28,29,30,31,32,33,34,35,36,37,38,39,40,41,42,43,44] included, 2 articles [22,41] were rated as excellent, and the other 21 articles [23,24,25,26,27,28,29,30,31,32,33,34,35,36,37,38,39,40,42,43,44] were categorized as good quality (Table 2).

### 3.3. The Characteristics of the Included Studies

Out of the 23 studies [22,23,24,25,26,27,28,29,30,31,32,33,34,35,36,37,38,39,40,41,42,43,44] included in this systematic review, 20 studies [22,23,24,25,26,28,29,30,33,34,35,36,37,38,39,40,41,42,43,44] involved males, with a total of 447 male participants, while 2 studies [27,31] involved females, with a total of 40 female participants, and 1 study [32] involved both males and females, including 13 male participants and 9 female participants. Participants in 18 studies [25,26,27,28,30,31,32,33,34,35,36,38,39,40,41,42,43,44] had experience with resistance training, while the remaining 5 studies [22,23,24,29,37] included participants with no experience of resistance training.

The doses of creatine used ranged from 15 to 25 g/day or 0.3 g/kg/day during the creatine loading period (i.e., the first ≤ 7 days of the creatine intervention) and from 2 to 10 g/day or from 0.03 to 0.22 g/kg/day during the remainder of the creatine intervention. The duration of the resistance training ranged from 4 to 12 weeks, with weekly frequencies ranging between two and five sessions.

Twenty-one studies provided data on upper-body muscle strength (*n* = 433) and nineteen studies provided data on lower-body muscle strength (*n* = 395), all of which were measurements of maximal muscle strength. Detailed information on the controlled trials included in this systematic review is presented in Table 3.

### 3.4. Meta-Analyses

#### 3.4.1. Upper-Body Muscle Strength

**Figure 2:**
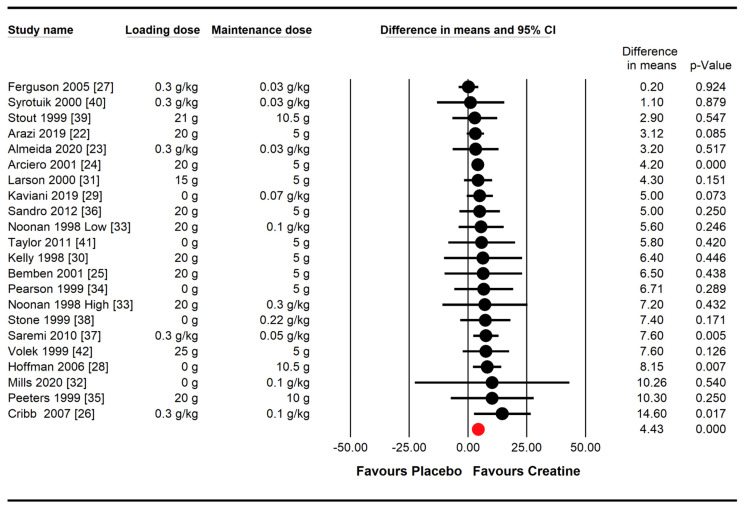
Effect of creatine supplementation and resistance training compared with a placebo and resistance training on upper-body strength. The red circle represents the pooled weighted mean difference following a random effect meta-analysis, expressed in kg.

Creatine supplementation combined with resistance training resulted in greater increases in upper-body strength compared with a placebo, with a very high probability (WMD = 4.43 kg, 95% CI [3.12,5.75], *p* < 0.001) (Figure 2). The test for heterogeneity showed that there was no statistically significant heterogeneity among studies for this outcome (*p* = 0.926, *I*^2^ = 0%).

**Figure 3:**
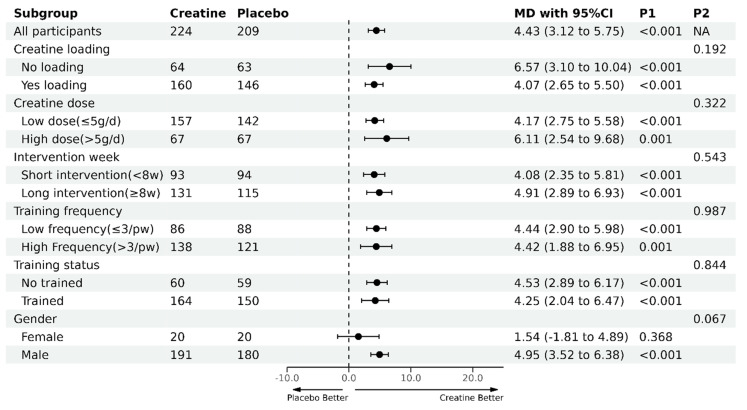
Subgroup analyses for creatine supplementation combined with resistance training on upper-body muscle strength compared with placebo supplementation combined with resistance training. MD, mean difference (kg); P1, *p*-value for the within-subgroup comparison (i.e., pre–post-intervention changes within each subgroup); P2, *p*-value for the between-subgroup comparison (i.e., comparison of the pre–post-intervention changes between subgroups); NA, Not Available.

Six subgroup meta-analyses were performed (Figure 3) according to the characteristics of the creatine supplementation protocol (the existence of creatine loading and creatine dose), the characteristics of the resistance training protocol (duration and frequency of training), and the characteristics of the participants (training status and sex).

Regarding biological sex differences, males on creatine experienced a greater improvement in upper-body strength compared with males on a placebo, with a very high probability (WMD = 4.95 kg, 95% CI [3.52, 6.38], *p* < 0.001). However, the effects of creatine did not reach statistical significance over a placebo in females (WMD = 1.54 kg, 95% CI [−1.81, 4.89], *p* = 0.368). There was a trend for greater upper-body strength gains from creatine in males vs. females (*p* = 0.067, Q = 3.366).

Creatine dosage (≤5 g/day vs. >5 g/day) and type of ingestion protocol, training status and duration and frequency of the training program had no influence on the upper-body strength gains obtained with creatine.

#### 3.4.2. Lower-Body Muscle Strength

**Figure 4:**
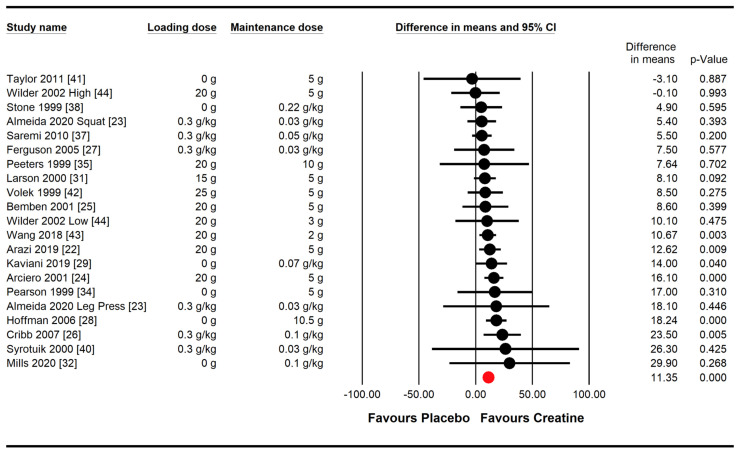
Effect of creatine supplementation and resistance training compared with a placebo and resistance training on lower-body muscle strength. The red circle represents the pooled weighted mean difference following a random effect meta-analysis, expressed in kg.

Creatine supplementation combined with resistance training produced greater increases in maximal lower-body strength compared with a placebo, with a very high probability (WMD = 11.35 kg, 95% CI [8.44,14.25], *p* < 0.001; Figure 4). Heterogeneity tests showed no statistically significant heterogeneity among studies for lower-body muscle strength gains (*p* = 0.897, *I*^2^ = 0%).

**Figure 5:**
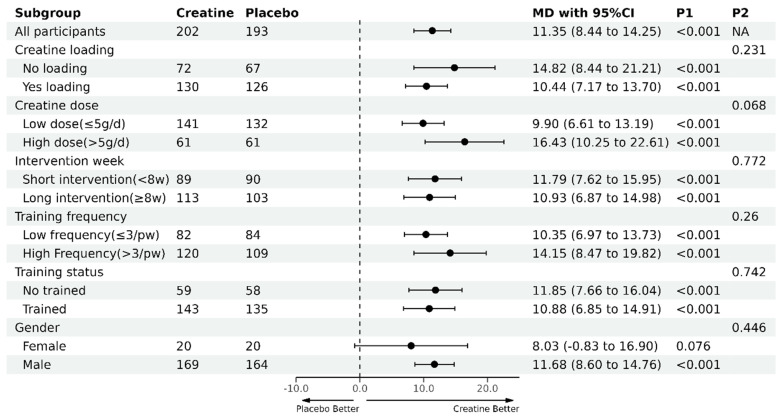
Subgroup analyses for creatine supplementation and resistance training compared with a placebo and resistance training on lower-body strength. MD, mean difference(kg); P1, *p*-value for the within-subgroup comparison (i.e., pre–post-intervention changes within each subgroup); P2, *p*-value for the between-subgroup comparison (i.e., comparison of the pre–post intervention changes between subgroups); NA, Not Available.

Similar to upper-body strength, six subgroup meta-analyses were analyzed for lower-body strength, including the same categories (Figure 5).

Regarding biological sex differences, males on creatine experienced a greater improvement in lower-body strength compared with males on a placebo, with very high probability (WMD = 11.68 kg, 95% CI [8.60,14.76], *p* < 0.001). However, creatine did not result in greater strength improvements compared with the placebo in females (WMD = 8.03 kg, 95% CI [−0.83,16.90], *p* = 0.076). Strength improvements from creatine were similar for males and females over time (*p* = 0.446, Q = 1.056).

With regard to dose–response differences, it is noteworthy that there was a trend (*p* = 0.068, Q = 3.341) indicating greater strength gains with high-dose creatine (>5 g/day) compared with lower-dose creatine (≤5 g/day) for lower-body strength gains. Creatine dosage (≤5 g/day vs. >5 g/day), ingestion protocol, training status, and the duration and frequency of training had no influence on strength gains between creatine and the placebo.

### 3.5. Publication Bias

**Figure 6:**
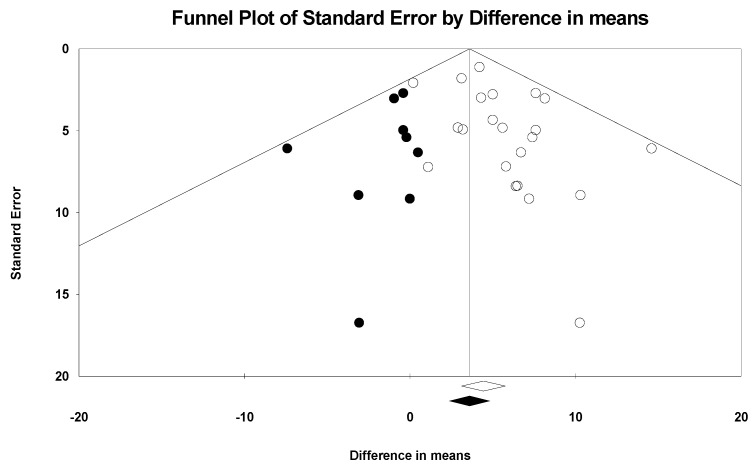
Observed and imputed funnel plot for upper-body muscle strength. The funnel plot displays the distribution of studies included in this meta-analysis, with white circles representing the original observed studies and black circles indicating the imputed studies added to account for potential publication bias using the trim and fill method. At the bottom of the funnel plot, the white and black diamonds represent the combined effect sizes, with the white diamond indicating the overall effect from the observed studies, and the black diamond showing the adjusted effect after imputation.

Funnel plots were used to detect publication bias. The funnel plot for the gains obtained with creatine over the placebo on upper-body muscle strength was asymmetric, suggesting possible publication bias. This was confirmed by Egger’s linear regression test (*t* = 2.102, *p* = 0.048). However, there was no significant change in upper-body muscle strength (WMD = 3.60 kg, 95% CI [2.39, 4.81]) after adjustment for Duval and Tweedie’s trim-and-fill test (Figure 6).

**Figure 7:**
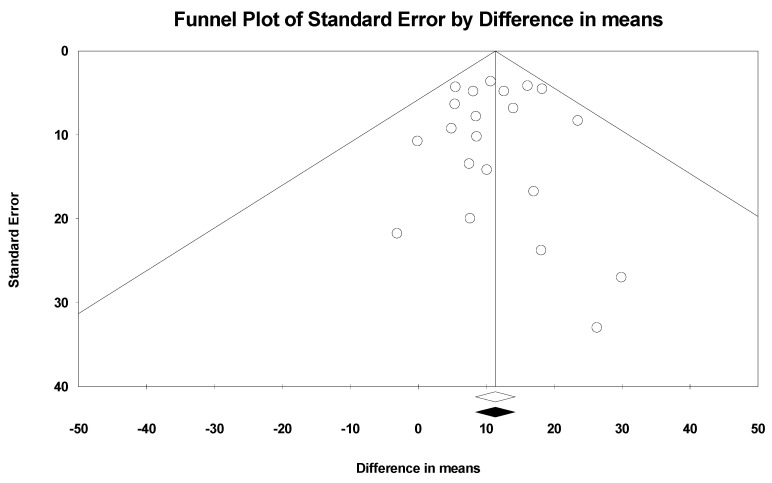
Observed and imputed funnel plot for lower-body muscle strength. The funnel plot displays the distribution of studies included in this meta-analysis, with white circles representing the original observed studies. At the bottom of the funnel plot, the white and black diamonds represent the combined effect sizes, with the white diamond indicating the overall effect from the observed studies and the black diamond showing the adjusted effect after imputation.

The funnel plot for the gains obtained with creatine over the placebo on lower-body muscle strength was symmetrical (Figure 7), indicating no significant publication bias, which was confirmed by Egger’s linear regression test (*t* = 0.122, *p* = 0.90).

## 4. Discussion

The most important findings from the meta-analyses were that: (1) the combination of creatine supplementation and resistance training results in greater strength gains in both the upper and lower body compared with resistance training alone in adults < 50 years of age, (2) males on creatine improved muscle strength compared with males on a placebo for both the upper and lower body, and (3) creatine had no significant effect on strength in females. Collectively, these results suggest that the addition of creatine supplementation to a resistance training program improves training adaptations for upper- and lower-body maximal muscle strength in healthy individuals under 50 years of age, and these effects are likely driven by the inclusion of biological males.

### 4.1. Effects of Creatine Supplementation on Muscle Strength

In the present meta-analysis, creatine supplementation during resistance training induced higher gains in maximal strength than placebo, with a 4.43 kg benefit for upper-body strength and an 11.35 kg benefit for lower-body strength. These results support previous meta-analyses [9,10,11,45] which showed that creatine supplementation increased measures of upper- and lower-body maximal strength. While no mechanisms were determined in any of these meta-analyses, it is possible that the greater improvements in muscle strength from creatine supplementation are related to creatine augmenting intramuscular creatine stores (i.e., PCr and free creatine), which would expedite ATP resynthesis and/or PCr recovery during and after exercise [46,47,48]. Additionally, creatine has also been shown to increase GLUT-4 transport kinetics, which could increase blood glucose disposal within the muscle and increase glycogen resynthesis [4,49,50]. Finally, while creatine is suggested to reduce blood lactate levels and enhance exercise performance [5], this study did not directly measure blood acidosis or H⁺ formation. The reduced lactate may indicate improved lactate utilization by muscle, potentially allowing individuals to perform more repetitions in each set, leading to greater muscle strength over time.

### 4.2. Characteristics of Creatine Supplementation

The most rapid way to increase muscle creatine stores is to ingest high doses (~20 g/day for 5–7 days; the creatine-loading phase) [51]. Once intramuscular creatine stores are fully saturated, muscle creatine stores can usually be maintained by ingesting small doses (~2–5 g/day) [3]. Another supplementation strategy is to refrain from the creatine loading phase and ingest lower daily doses (~3–5 g/day) of creatine monohydrate for longer periods of time (i.e., ≥28 days) [52]. The results showed no significant difference in strength gains between lower-dose (≤5 g/d) vs. high-dose(>5 g/d) creatine, with gains of 4.17 kg vs. 6.11 kg for upper-body strength, respectively. Although the lower-dose supplementation approach may delay the saturation of intramuscular creatine levels [52], the intervention cycles of the RCTs analyzed were all ≥ 4 weeks, which may account for the lack of significance between the lower- and high-dose creatine protocols. Therefore, it is likely that the presence of a creatine loading phase may not be needed if the objective is to increase muscle strength when performing resistance training for ≥ 4 weeks. Specifically, the results of this study show that for upper-body strength, the subgroup of studies including a loading phase had an average increase of 6.57 kg, while the subgroup of studies without a loading phase had an increase of 4.07 kg; for lower body strength, the loading subgroup increased by 14.82 kg compared with 10.44 kg for the non-loading subgroup. Although the comparison between the two methods of supplementation with creatine did not report statistically significant differences, the slightly higher muscle strength gains in those studies with loading vs. non-loading phases (2.50 kg for the upper body and 4.38 kg for lower-body strength) may be considered as potentially beneficial by some strength training practitioners, especially if seeking fast strength gains with resistance training programs shorter than 4 weeks.

Regarding possible creatine dose–response differences, the results showed that high-dose creatine supplementation (>5 g/day) had a favorable but not significantly larger effect (9.90 kg vs. 16.43 kg for the lower body, *p* = 0.068) on lower-limb muscle strength, compared with lower-dose creatine (≤5 g/day). This is similar to another previous meta-analysis with a similar approach [45]. This may be due to the fact that individuals with a heavier body weight may need to consume as much as 5–10 g/day of creatine in order to maintain stores [53,54], making the enhancement effect of higher doses of creatine supplementation (>5 g/day) significant. Collectively, all this information suggests that the loading phase and the dose are not key factors to obtain further strength gains with creatine supplementation combined with resistance training. However, these factors (i.e., including a loading phase or the use of doses ≤ 5 g/day) may make valuable contributions for shorter resistance training programs or for those focused on the lower limbs.

### 4.3. Population Characteristics

Regarding biological sex differences, the present meta-analysis showed that creatine supplementation + resistance training demonstrated superior efficacy for improving muscle strength compared with a placebo and resistance training in males only, with average increases of 4.95 kg in upper-body strength and 11.68 kg in lower-body strength. In contrast, the analysis found that creatine supplementation combined with resistance training did not result in a significant improvement in muscle strength for females, with average increases of 1.54 kg in upper-body strength and 8.03 kg in lower-body strength, indicating no notable differences compared with the placebo and resistance training alone. Mechanistically, the blunted response in females may be related to higher pre-supplementation intramuscular creatine levels [55], which may attenuate the responsiveness to creatine supplementation over time [56]. There is also some evidence that females do not experience a reduction in measures of amino acid catabolism to the same extent as males [57]. Further, menstrual cycle fluctuations and estrogen cessation cannot be ruled out [56].

### 4.4. Characteristics of Resistance Training

Subgroup analyses showed that the duration of training did not influence strength adaptations over time, with average increases of 4.08 kg for upper-body strength in the short-duration subgroup compared with 4.91 kg in the long-duration subgroup, and 11.79 kg in the short-duration subgroup compared with 10.93 kg in the long-duration subgroup for lower-body strength. These results are in contrast to a previous review [11], which showed that interventions of ≥24 weeks appeared to be more effective in resistance-trained older females. Unfortunately, the studies included in this investigation with healthy participants < 50 years or age included resistance training protocols with a duration ranging from 4 to 12 weeks. Therefore, it was not possible to determine the optimal duration of an RT for younger populations aiming to maximize the strength gains obtained with creatine supplementation. This study reveals the need to experiment with the effects of creatine supplementation during RT for more than 12 weeks in young and healthy adults, as has been carried out in other populations. Additionally, different resistance training frequencies seemed to not affect the adaptation of creatine supplementation combined with resistance training to muscle strength, with average increases of 4.44 kg for upper-body strength in the low-frequency subgroup compared with 4.42 kg in the high-frequency subgroup, and 10.35 kg in the low-frequency subgroup compared with 10.93 kg in the high-frequency subgroup for lower-body strength. These data should be interpreted cautiously, as the studies had different durations and frequencies, and even the subgroups created to normalize training frequency presented studies with a certain variation.

### 4.5. Limitations, Practical Applications, and Future Research Directions

Several limitations must be acknowledged in this study.

Firstly, despite applying selective search and inclusion filters, the included studies utilized varying doses of creatine and differing durations of supplementation, and some included creatine-loading phases, while others did not. In addition, nine studies adjusted the doses based on the participants’ body mass, whereas others used absolute doses of creatine. These variations likely introduced differences in the magnitude of creatine’s effects compared with a placebo [58,59] and may have led to publication bias regarding creatine’s impact on upper-body muscle strength. Secondly, the intensity, volume, and types of resistance training protocols varied across included studies, preventing the determination of which type of resistance training optimally enhances muscle strength. Thirdly, the effects of creatine supplementation combined with resistance training on different sexes should be interpreted with caution, as only two studies included female participants. Lastly, other factors, such as the timing of creatine supplementation and its combination with substances such as CHO were not analyzed, though these factors could influence muscle creatine uptake and, consequently, the benefits obtained from supplementation.

Despite its limitations, this research provides valuable insights for resistance training enthusiasts and offers a direction for future studies. The findings suggest that creatine supplementation, with or without a loading phase, can significantly enhance the strength gains from resistance training. This indicates that resistance training enthusiasts may experience faster and greater strength improvements with creatine supplementation compared with training without it. Additionally, this investigation also suggests that, overall, all doses of creatine lead to benefits, but higher doses of creatine (greater than 5 g/day) may lead to more substantial improvements in lower-body strength. Therefore, it is advisable to adjust the creatine dosage on the basis of individual body weight during resistance training, while some athletes focused on lower-body strength may consider the use of higher doses of creatine. The current findings suggest that the periodization of resistance training and training frequency have minimal effects on strength adaptations related to creatine supplementation. This outcome may be a conclusion affected by the different training protocols used and the relatively small number of studies that used comparable strength training protocols. Future investigations should aim to study the effect of creatine supplementation with modifications of the intensity, frequency, time, and type of resistance training. These future investigations should focus on resolving which strength training protocol maximizes the benefits that can be obtained with creatine. Finally, it is crucial to explore potential sex differences in these responses, as there is a limited number of studies focused on female participants.

## 5. Conclusions

Creatine supplementation combined with resistance training significantly improves upper- and lower-body muscle strength compared with resistance training alone in healthy individuals < 50 years of age. Specifically, the data on this systematic review and meta-analysis suggest that 4–12 weeks of 2 to 10 g/day or from 0.03 to 0.22 g/kg/day of creatine supplementation combined with resistance training significantly improves upper-body muscle strength by 4.43 kg and lower-body muscle strength by 11.35 kg compared with resistance training alone in healthy individuals < 50 years of age. The benefits of creatine supplementation combined with resistance training are likely of higher magnitude in males than in females. Future research should prioritize investigating different resistance training protocols to determine which approach maximizes the benefits of creatine supplementation. Additionally, addressing the imbalance in male and female representation is crucial for developing more generalizable supplementation strategies.

## Figures and Tables

**Table 1:** PICOS criteria for the inclusion of RCTs in which the supplementation of creatine was combined with a well-structured resistance training program and pre–post-training strength gain was compared with placebo supplementation with resistance training.

Parameters	Inclusion	Exclusion
Population	Healthy individuals under the age of 50	Individuals with diseases or those over the age of 50.
Intervention	Creatine supplementation with structured resistance training	Creatine supplementation with other types of training (e.g., aerobic training), with unstructured training or without any type of training
Comparison	Placebo with the same structured resistance training as the intervention group	Placebo without resistance training or resistance training without a placebo
Outcomes	Muscle strength gains	Any other form of performance or anthropometric assessments
Study design	Randomized controlled trials (RCTs)	Meta-analyses, articles without full text, acute interventions, non-peer-reviewed articles, opinion pieces, reviews, case reports, and editorials

**Table 2:** Evaluation of the methodological quality of eligible studies (*n* = 23) utilizing the Physiotherapy Evidence Database (PEDro) scale.

	Assessment Criteria												
Study	1	2	3	4	5	6	7	8	9	10	11	Total Score	Quality Assessment
Almeida 2020 [23]	Y	1	0	1	1	1	0	1	0	1	1	7	Good
Arazi 2019 [22]	Y	1	1	1	1	1	1	1	0	1	1	9	Excellent
Arciero 2001 [24]	Y	1	0	1	1	1	0	1	0	1	1	7	Good
Bemben 2001[25]	Y	1	0	1	1	1	0	1	0	1	1	7	Good
Cribb 2007 [26]	Y	1	0	1	1	1	0	0	0	1	1	6	Good
Ferguson 2005 [27]	Y	1	0	1	1	1	0	0	0	1	1	6	Good
Hoffman 2006 [28]	Y	1	1	1	1	1	0	1	0	1	1	8	Good
Kaviani 2019 [29]	Y	1	0	1	1	1	0	0	1	1	1	7	Good
Kelly 1998 [30]	Y	1	0	1	1	1	0	1	0	1	1	7	Good
Larson 2000 [31]	Y	1	1	0	1	1	0	0	0	1	1	6	Good
Mills 2020 [32]	Y	1	1	1	1	1	0	0	1	1	1	8	Good
Noonan 1998 [33]	Y	1	1	1	1	1	0	0	1	1	1	8	Good
Pearson 1999 [34]	Y	1	0	1	1	1	0	1	0	1	1	7	Good
Peeters 1999 [35]	N	1	0	1	1	1	0	0	0	1	1	6	Good
Sandro 2012 [36]	Y	0	1	0	1	1	0	1	0	1	1	6	Good
Saremi 2010 [37]	Y	1	0	1	1	1	0	0	0	1	1	6	Good
Stone 1999 [38]	Y	1	0	1	1	1	0	1	0	1	1	7	Good
Stout 1999 [39]	Y	1	0	1	1	1	0	0	0	1	1	6	Good
Syrotuik 2000 [40]	Y	1	0	1	1	1	0	0	0	1	1	6	Good
Taylor 2011 [41]	Y	1	1	1	1	1	0	1	1	1	1	9	Excellent
Volek 1999 [42]	Y	1	0	1	1	1	0	1	0	1	1	7	Good
Wang 2018 [43]	Y	1	1	1	1	1	0	1	0	1	1	8	Good
Wilder 2002 [44]	Y	1	0	1	1	0	0	1	1	1	1	7	Good

1, eligibility criteria; 2, random allocation; 3, concealed allocation; 4, baseline comparability; 5, blinded subjects; 6, blinded therapists; 7, blinded assessors; 8, adequate follow-up; 9, intention-to-treat analysis; 10, between-group comparisons; 11, point estimates and variability. The total score represents the score of the PEDro scale. Item 1 was not scored. Y, yes; N, no.

**Table 3:** Information on the studies that were included in the systematic review (*n* = 23).

Author, Year, Country	Participants, Total N, Age	Duration, Sessions/ Week	Creatine, Loading Protocol	Creatine, Maintenance Protocol	Muscle Group Location
Almeida et al. [23], 2020, Brazil	Untrained males, 34, 23.45 ± 3.17	4 weeks, 3/week	0.3 g/kg/day for 7 days	0.03 g/kg/day for 21 days	Upper body, lower body
Arazi et al. [22], 2019, Iran	Untrained males, 16, 18 ± 3	6 weeks, 3/week	20 g/day for 5 days	5 g/day for 32 days	Upper body, lower body
Arciero et al. [24], 2001, USA	Untrained males, 20, 22 ± 2.99	4 weeks 3/week	20 g/day for 5 days	5 g/day for 23 days	upper body, lower body
Bemben et al. [25], 2001, USA	Trained males, 17, 19.35 ± 0.34	9 weeks 4/week	20 g/day for 5 days	5 g/day for 44 days	Upper body, lower body
Cribb et al. [26], 2007, Australia	Trained males, 15, 24.53 ± 6.27	11 weeks 4/week	0.3 g/kg/day for 7 days	0.1 g/kg/day for 70 days	Upper body, lower body
Ferguson et al. [27], 2005, Canada	Trained females, 26, 24.6 ± 3.68	9.5 weeks 4/week	0.3 g/kg/day for 7 days	0.03 g/kg/day for 58 days	Upper body, lower body
Hoffman et al. [28], 2006, USA	Trained males, 33, NR	10 weeks 4/week	No loading	10.5 g/day for 70 days	Upper body, lower body
Kaviani et al. [29], 2019, Canada	Untrained males, 18, 23 ± 3	8 weeks 3/week	No loading	0.07 g/kg/day for 56 days	Upper body, lower body
Kelly et al. [30], 1998, Australia	Trianed males, 18, 26.8 ± 5.78	4 weeks 2/week	20 g/day for 5 days	5 g/day for 21 days	Upper body
Larson et al. [31], 2000, USA	Trained females, 14, 19.15 ± 1.40	12 weeks 3/week	15 g/day for 5 days	5 g/day for 12 weeks	Upper body, lower body
Mills et al. [32], 2020, USA	Trained males and females, 22, 26 ± 4.32	6 weeks 5/week	No loading	0.1 g/kg/day for 6 weeks	Upper body, lower body
Noonan et al. [33], 1998, USA	Trained males, 39, 19.83 ± 1.3	8 weeks 4/week	20 g/day for 5 days	Low dose: 0.1 g/kg/day for 51 days High dose: 0.3 g/kg/day for 51 days	Upper body
Pearson et al. [34], 1999, USA	Trained males, 16, 20.7	10 weeks 4/week	No loading	5 g/day for 70 days	Upper body, lower body
Peeters et al. [35], 1999, USA	Trianed males, 34, 21.2 ± 2.6	6 weeks 4/week	20 g/day for 3 days	10 g/day for 39 days	Upper body, lower body
Sandro et al. [36], 2012, Brazil	Trained males 18, 17.10 ± 1.63	4 weeks 3/week	20 g/day for 5 days	5 g/day for 27 days	Upper body
Saremi et al. [37], 2010, Iran	Untrained males, 16, 23.06 ± 2.65	8 weeks 3/weeks	0.3 g/kg/day for 7 days	0.05 g/kg/day for 35 days	Upper body, lower body
Stone et al. [38], 1999, USA	Trained males, 20, 18.48 ± 0.74	5 weeks 3/week	No loading	0.22 g/kg/day for 35 days	Upper body, lower body
Stout et al. [39], 1999, USA	Trained males, 16, 19.6 ± 1.0	8 week 4/week	21 g/day for 5 days	10.5 g/day for 51 days	Upper body
Syrotuik et al. [40], 2000, Canada	Trained males, 14, 23.15 ± 0.89	5 weeks 4/week	0.3 g/kg/day for 5 days	0.03 g/kg/day for 32 days	Upper body, lower body
Taylor et al. [41], 2011, USA	Trained males, 29, 20.38 ± 2.88	8 week 4/week	No loading	5 g/day for 8 weeks	Upper body, lower body
Volek et al. [42], 1999, USA	Trained males, 19, 25.51 ± 5.2	12 weeks 4/week	25 g/day for 7 days	5 g/day for 77 days	Upper body, lower body
Wang et al. [43], 2018, China	Trained males, 30, 20 ± 1.55	4 week 3/week	20 g/day for 6 days	2 g/day for 22 days	Lower body
Wilder et al. [44], 2002, USA	Trained males, 25, 19 ± 1.02	10 week 4/week	20 g/day for 1 week	Low dose: 3 g/day for 10 weeks High dose: 5 g/day for 9 weeks	Lower body

## Data Availability

The raw data supporting the conclusions of this article will be made available by the authors on request. The data are not publicly available due to concerns regarding data integrity.
